# Supplementary material for: Brown adipose tissue CoQ deficiency activates the integrated stress response and FGF21-dependent mitohormesis
Source: EMBO J. 2024 Jan 11;43(2):2. doi: 10.1038/s44318-023-00008-x (PMC10897314; doi:10.1038/s44318-023-00008-x)
Supplement: Supplementary file 1 — Appendix [file 44318_2023_8_MOESM1_ESM.pdf]

Appendix

Table of Contents

Supplementary Figures

Appendix Figure S1: ATF4 induces components crucial in metabolic adaptation by BAT CoQ-deficiency ……………………………………………………………….2-3

Appendix Figure S2: Characterizing FGF21 effects on BAT and peripheral tissues ………………………………………………………………………………………………4-5

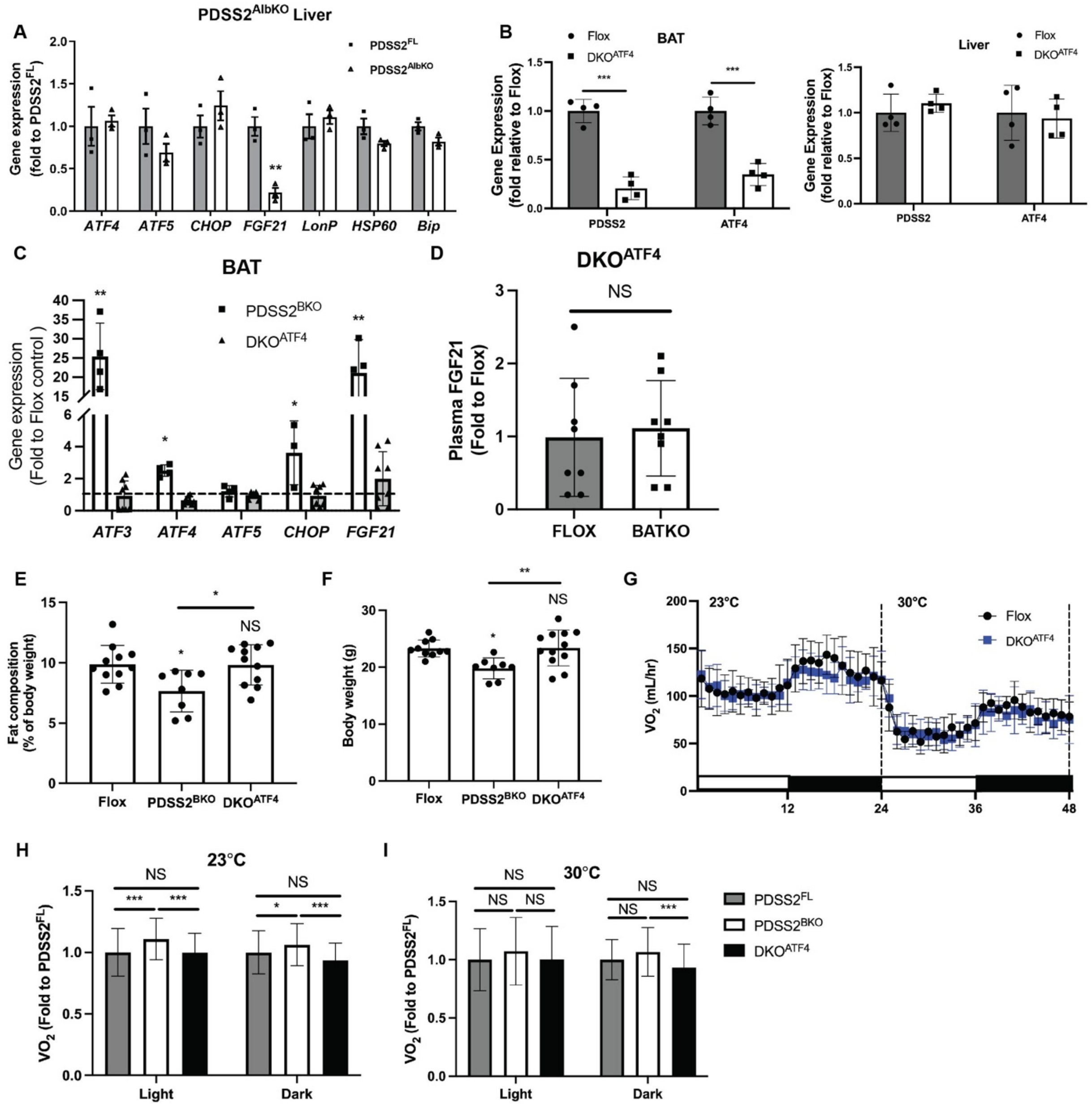

Appendix Figure S1. ATF4 induces components crucial in metabolic adaptation by BAT CoQ-deficiency

A Stress-related gene expression in liver of PDSS2^{FL} or liver-specific PDSS2 knockouts (PDSS2^{AlbKO}), n=3.

B Validation of PDSS2 and ATF4 via qPCR analysis of BAT (left panel) and liver (right panel) of floxed controls (Flox) and BAT-specific PDSS2 and ATF4 double knockout animals (DKO^{ATF4}) animals, n=4.

C ISR-related gene expression in BAT of PDSS2^{BKO} and DKO^{ATF4} mice, n=4-8.

D Plasma FGF21 levels of DKO^{ATF4} compared to floxed controls, n=8.

E-F Body weight and fat composition of control Flox, PDSS2^{BKO} and DKO^{ATF4} after 2-week defined diet at age 10-week, n=8-11.

G Oxygen consumption rate of PDSS2^{FL} and DKO^{ATF4} animals was monitored under 23 and 30°C in real-time in metabolic cages using CLAMS, n=5.

H-I Averaged VO_2 of PDSS2^{FL}, PDSS2^{BKO} and DKO^{ATF4} at 23 and 30°C, n=5-11.

Data information: Data are mean ± SEM. (A-D) Results were compared using an unpaired two-tailed student t test. (E-F, H-I) Results were compared using a one-way ANOVA test. Significance presented at P*<0.05, P**<0.01, and P***<0.001 compared to controls. Data describes biological replicates.

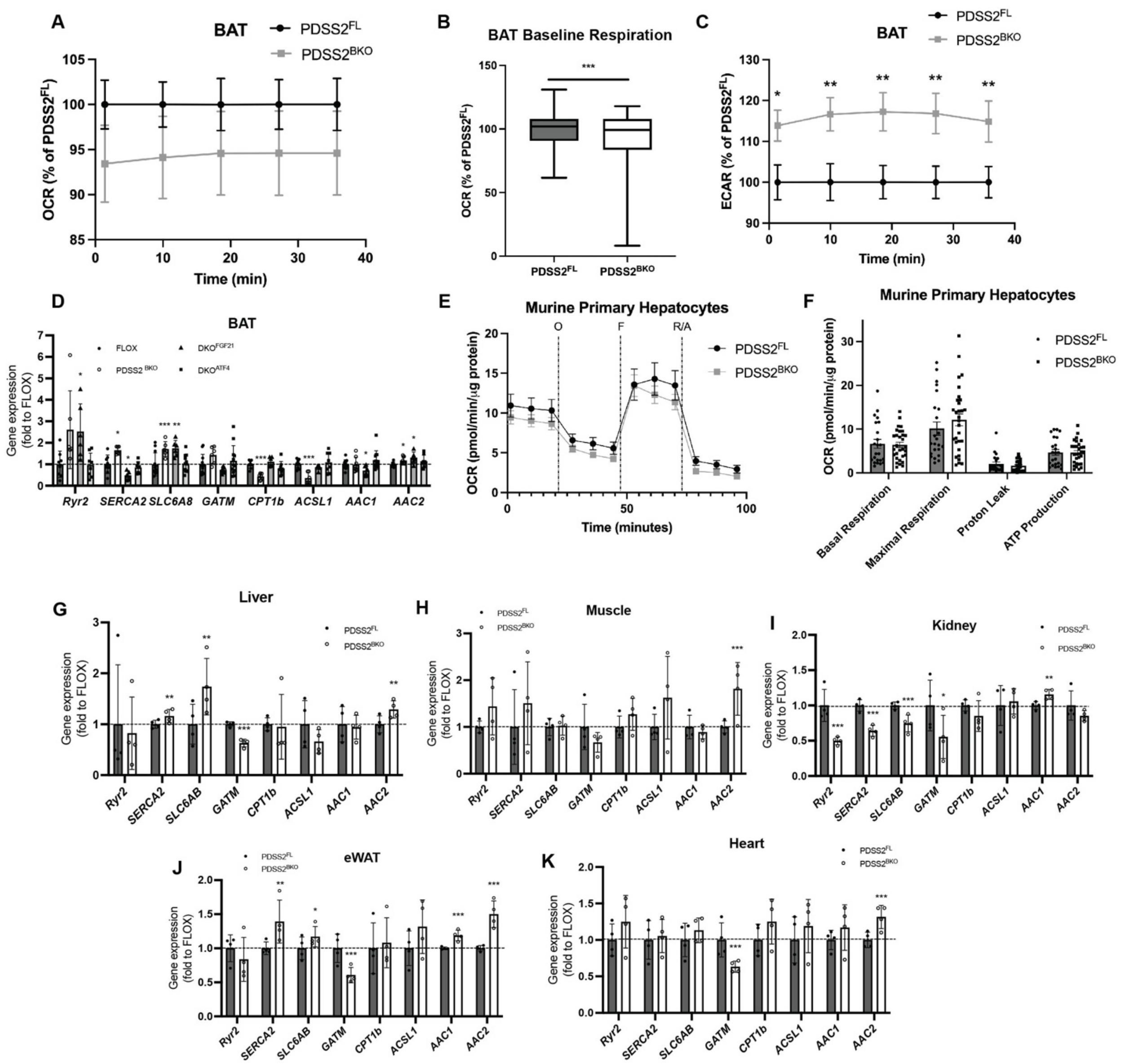

Appendix Figure S2. Characterizing FGF21 effects on BAT and peripheral tissues

A OCR of BAT tissue pieces from PDSS2^{FL} or PDSS2^{BKO} mice. BAT was dissected from 5 mice per group with 5 tissue pieces analyzed per mouse for a total of 25 tissue pieces analyzed per group.

B Baseline respiration of BAT tissue pieces. Data from the 5 timepoints from the OCR trace in A were pooled and compared between PDSS2^{FL} or PDSS2^{BKO} BAT pieces. Centerline represents median and box extends from 25th to 75th percentiles. BAT was dissected from 5 mice per group with 5 tissue pieces analyzed per mouse for a total of 25 tissue pieces analyzed per group.

C Extracellular acidification rate (ECAR) of BAT tissue pieces from PDSS2^{FL} or PDSS2^{BKO} mice. BAT was dissected from 5 mice per group with 5 tissue pieces analyzed per mouse for a total of 25 tissue pieces analyzed per group.

D Genes of UCP1-independent thermogenesis in BAT of BAT-specific PDSS2, ATF4 and PDSS2, or FGF21 and PDSS2 knockouts ($PDSS2^{BKO}$, DKO^{ATF4} and DKO^{FGF21}), n=6-8/group.

E OCR of murine primary hepatocytes isolated from $PDSS2^{FL}$ or $PDSS2^{BKO}$ mice. Hepatocytes were isolated from 3 mice per group and plated in 5-10 wells per mouse.

F Basal, maximal, proton leak and ATP production respiration of primary hepatocytes isolated from $PDSS2^{FL}$ or $PDSS2^{BKO}$ mice, n=3 mice/group.

G-K Genes of UCP1-independent thermogenesis in liver, muscle, kidney, eWAT or heart of $PDSS2^{FL}$ and $PDSS2^{BKO}$ animals, n=4.

Data information: All data except panel B are mean ± SEM. Results were compared using an unpaired two-tailed student t test. Significance presented at P*<0.05, P**<0.01, and P***<0.001 compared to controls. Data describes biological replicates.
