# Supplementary figures and images for: Brown adipose tissue CoQ deficiency activates the integrated stress response and FGF21-dependent mitohormesis
Source: EMBO J. 2024 Jan 11;43(2):2. doi: 10.1038/s44318-023-00008-x (PMC10897314; doi:10.1038/s44318-023-00008-x)

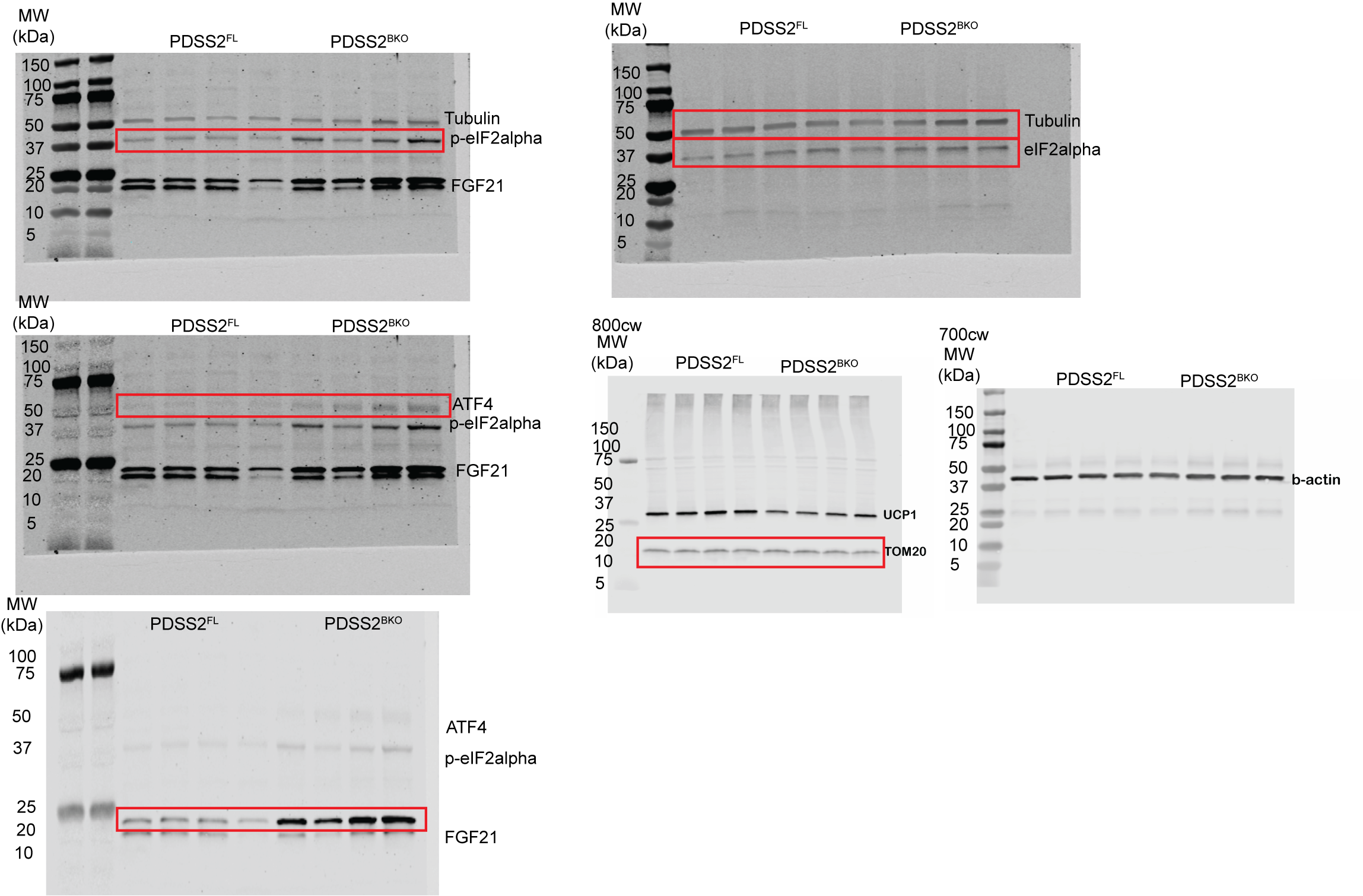

Supplement: Supplementary file 2 — Source Data Fig. 1 [file 44318_2023_8_MOESM2_ESM.zip › Figure 1/1G/western stress factors.tif]

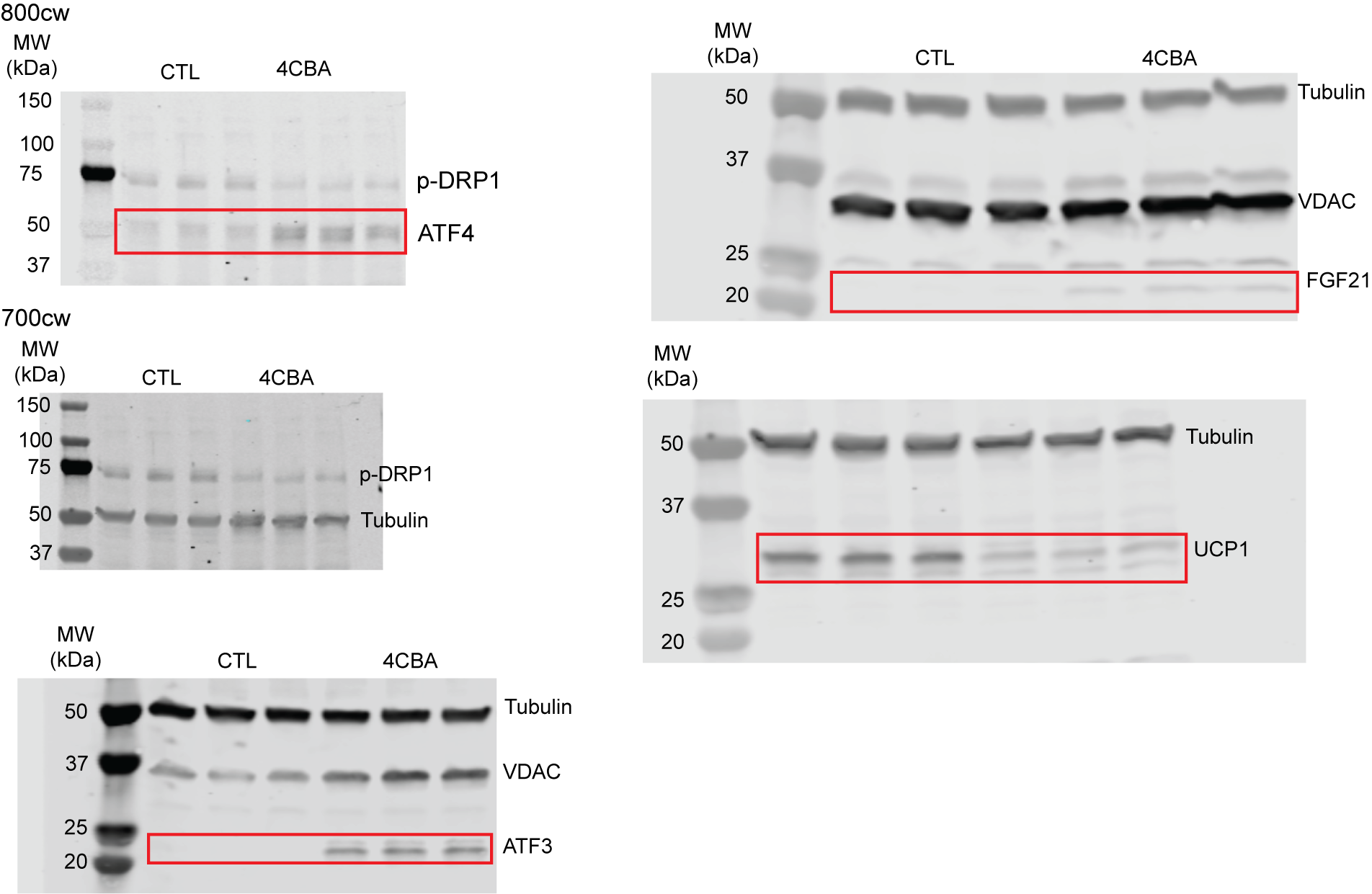

Supplement: Supplementary file 2 — Source Data Fig. 1 [file 44318_2023_8_MOESM2_ESM.zip › Figure 1/1E/western ucp1 and stress factors.tif]

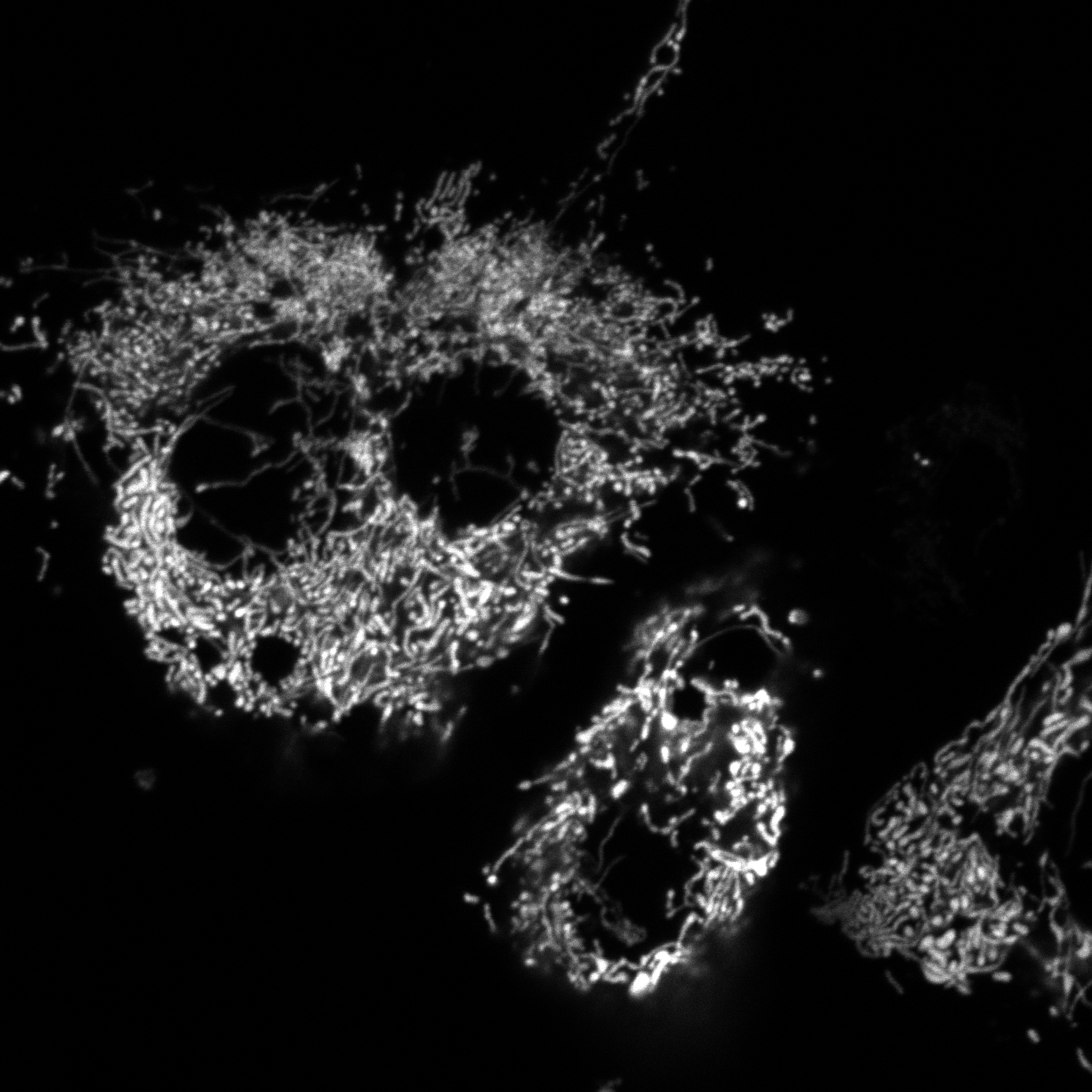

Supplement: Supplementary file 3 — Source Data Fig. 2 [file 44318_2023_8_MOESM3_ESM.zip › Figure 2/2A/confocul mito LD nuc CTL.tif]

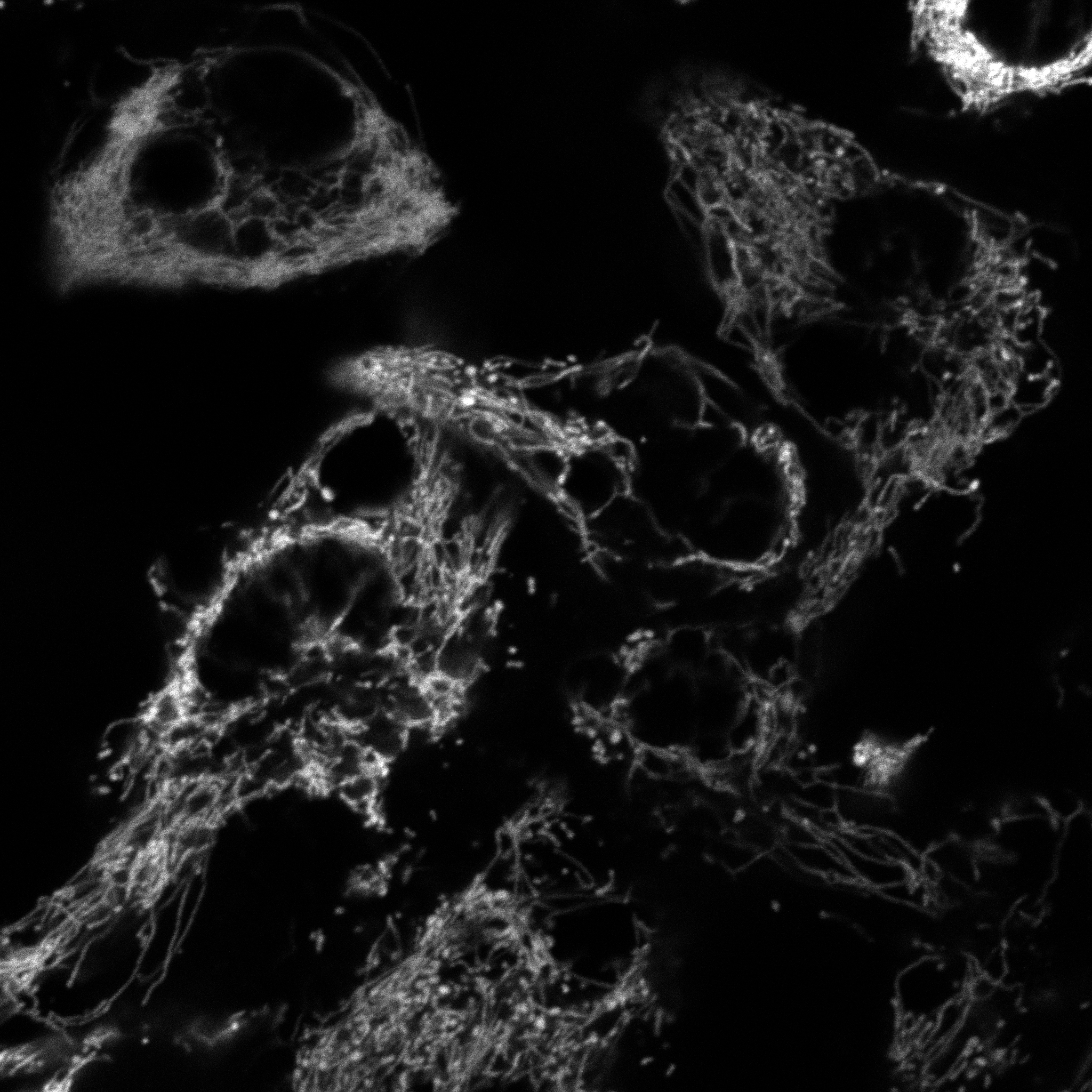

Supplement: Supplementary file 3 — Source Data Fig. 2 [file 44318_2023_8_MOESM3_ESM.zip › Figure 2/2A/confocul mito LD nuc 4CBA.tif]

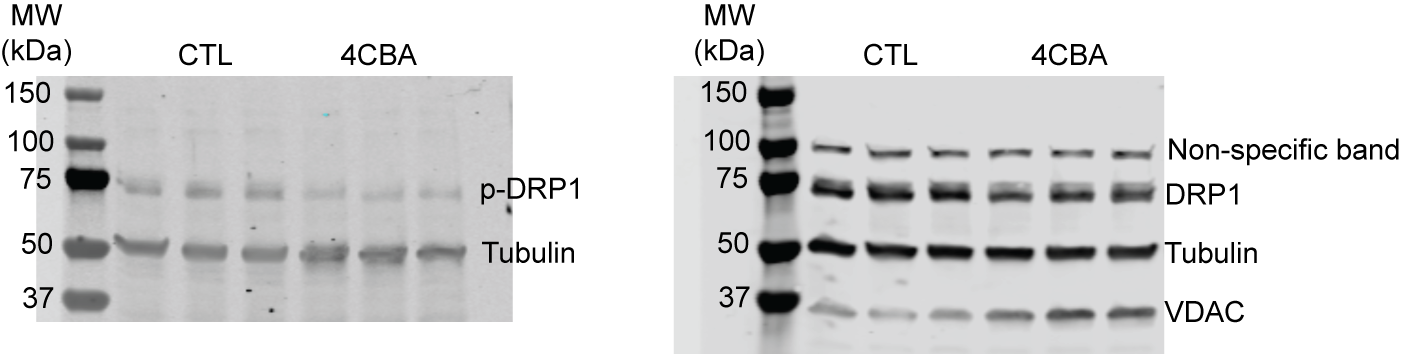

Supplement: Supplementary file 3 — Source Data Fig. 2 [file 44318_2023_8_MOESM3_ESM.zip › Figure 2/2C/western pDRP1 DRP1 VDAC.tif]

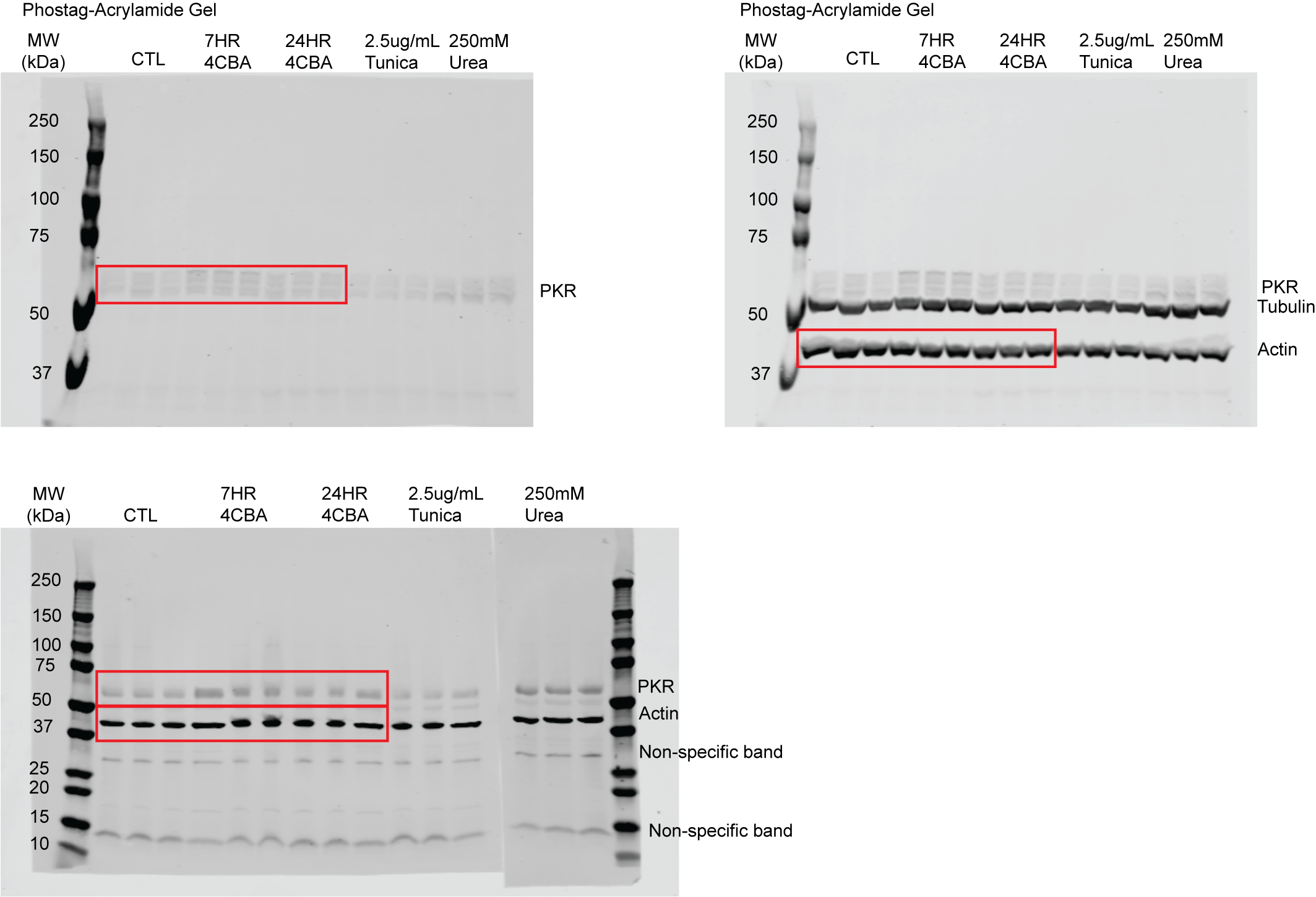

Supplement: Supplementary file 4 — Source Data Fig. 3 [file 44318_2023_8_MOESM4_ESM.zip › Figure 3/3C/western phosphopkr pkr.tif]

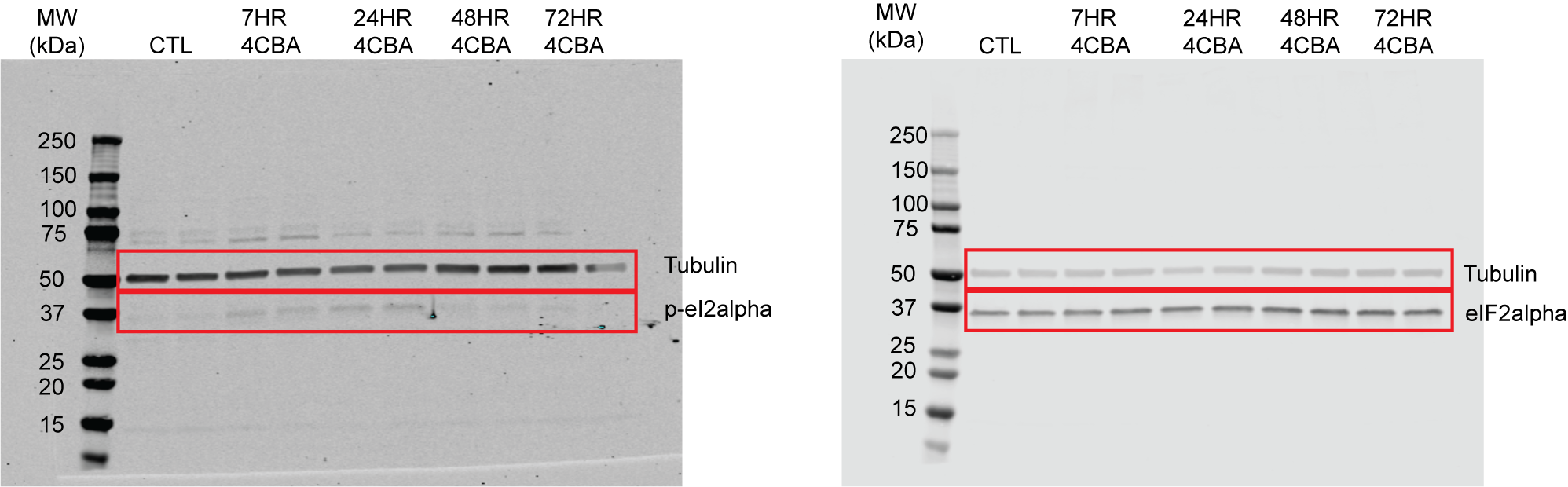

Supplement: Supplementary file 5 — Source Data Fig. 4 [file 44318_2023_8_MOESM5_ESM.zip › Figure 4/4B/western peif2a_eif2a.tif]

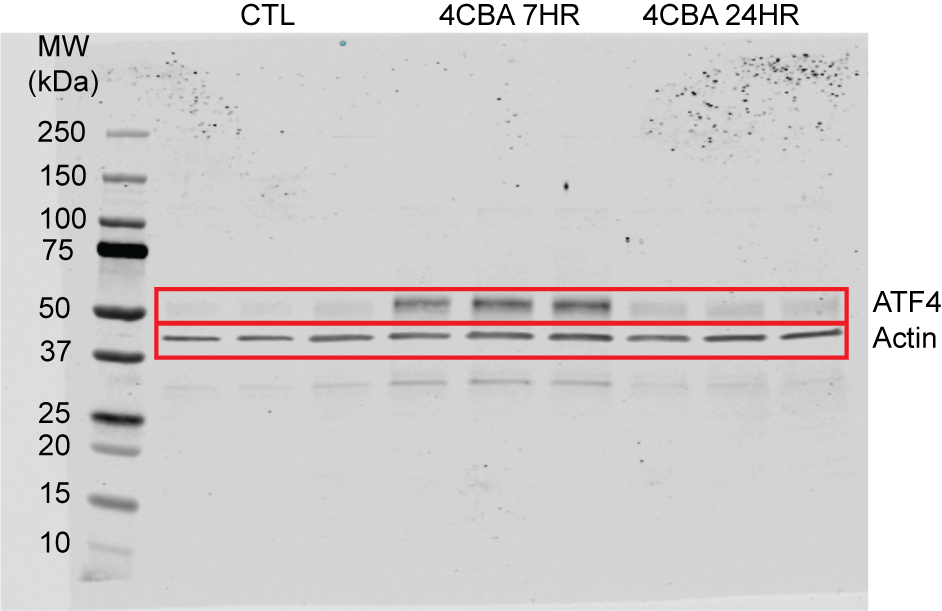

Supplement: Supplementary file 5 — Source Data Fig. 4 [file 44318_2023_8_MOESM5_ESM.zip › Figure 4/4C/western atf4.tif]

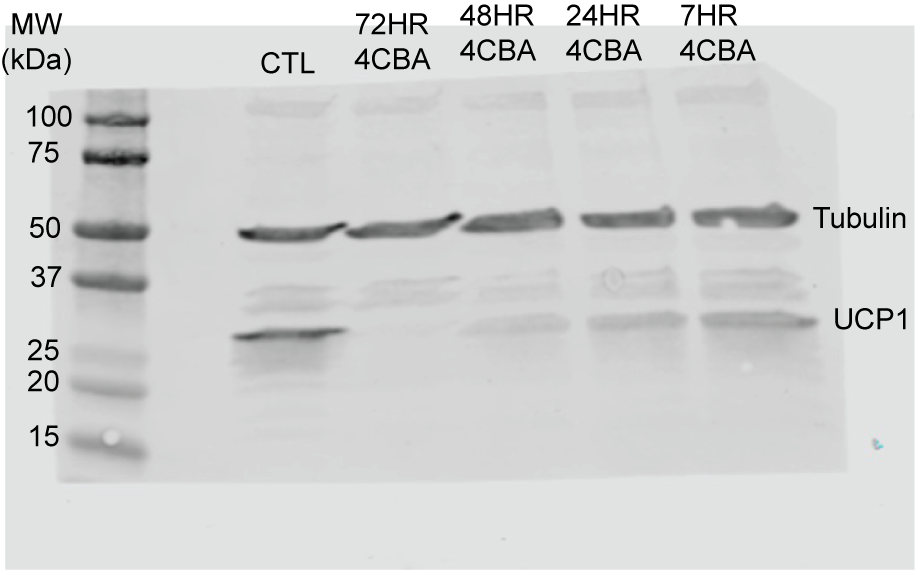

Supplement: Supplementary file 5 — Source Data Fig. 4 [file 44318_2023_8_MOESM5_ESM.zip › Figure 4/4H/western UCP1.tif]

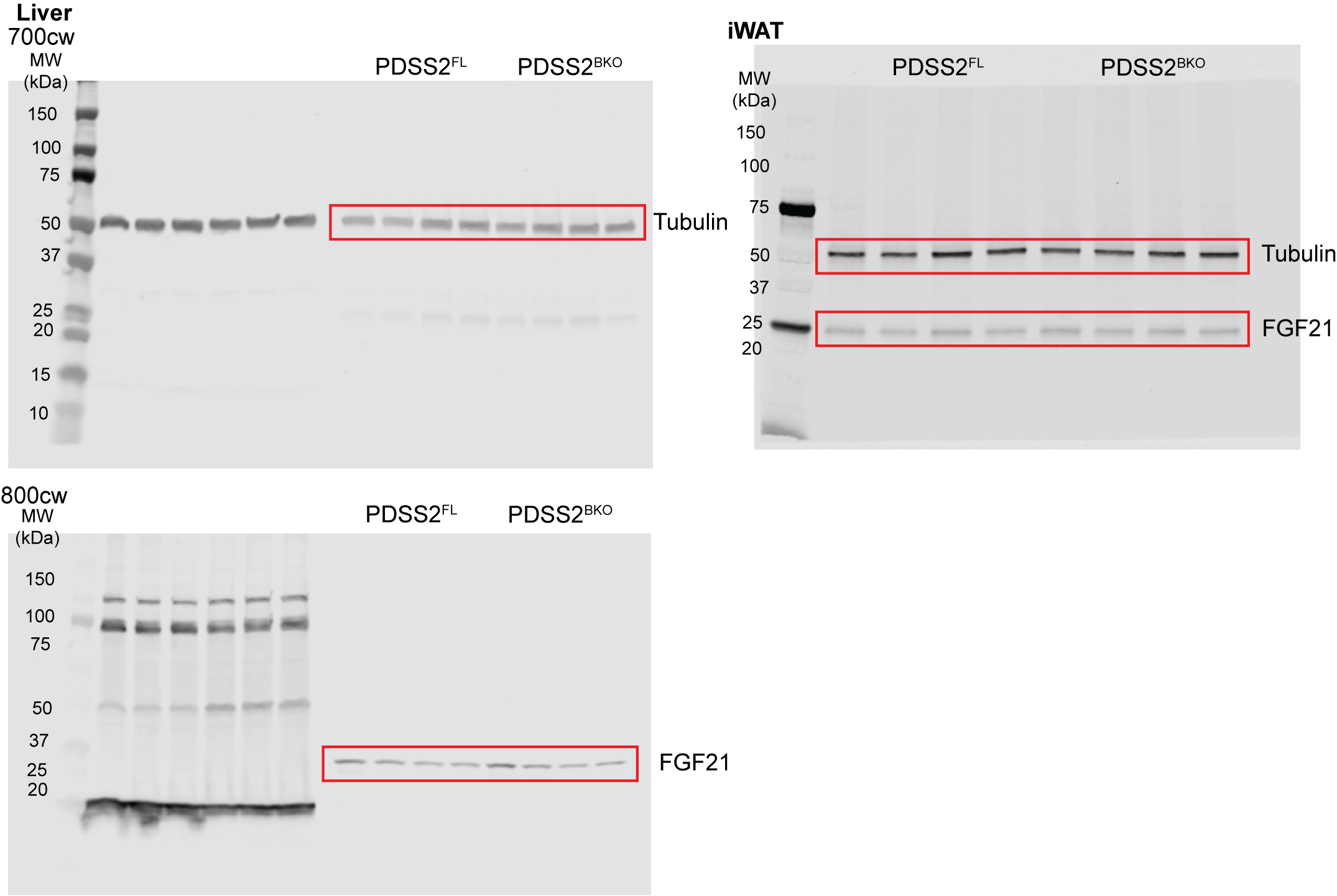

Supplement: Supplementary file 7 — Source Data Fig. 6 [file 44318_2023_8_MOESM7_ESM.zip › Figure 6/6E/western FGF21.tif]

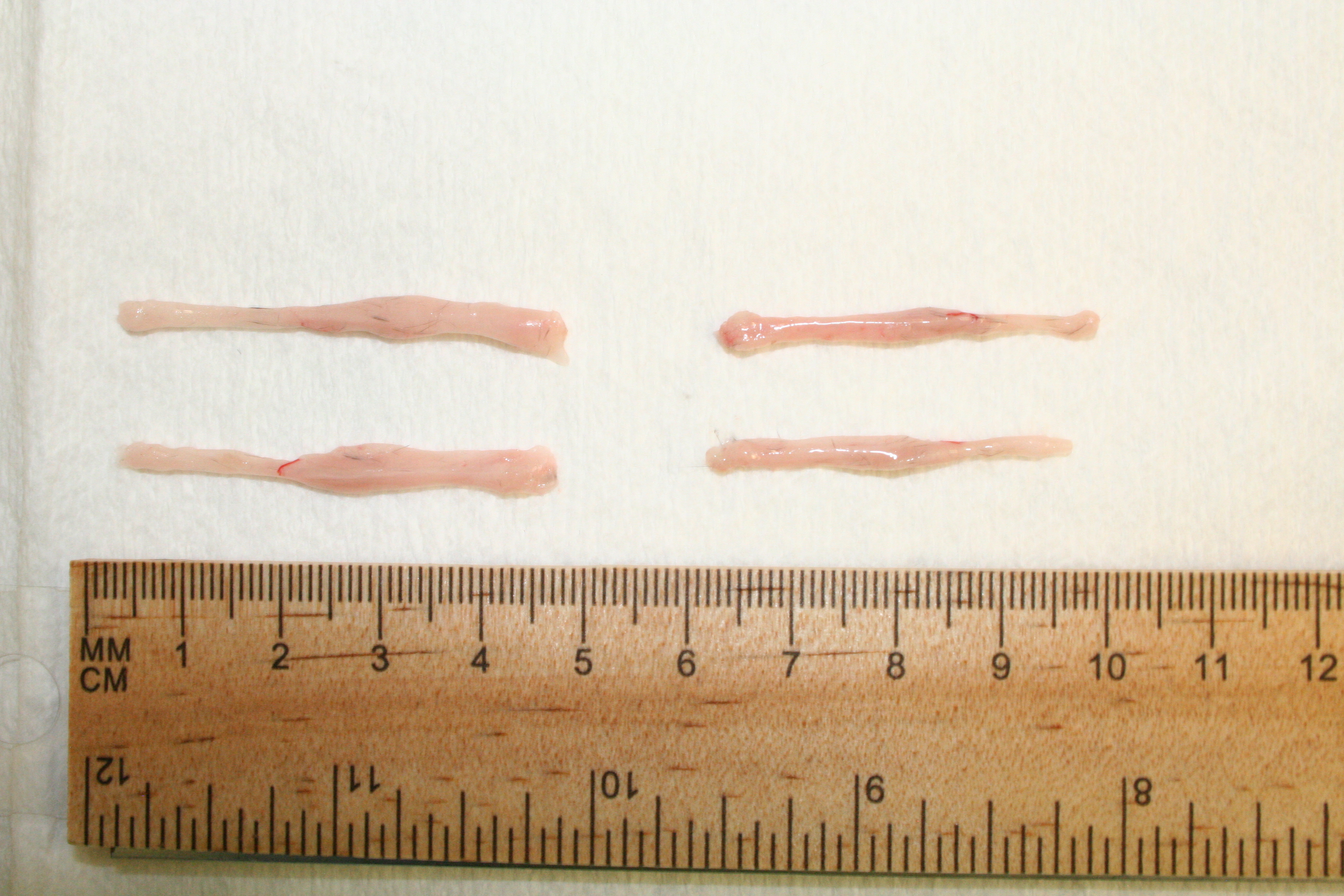

Supplement: Supplementary file 8 — Source Data Fig. 7 [file 44318_2023_8_MOESM8_ESM.zip › Figure 7/7D/Image iWAT.tiff]

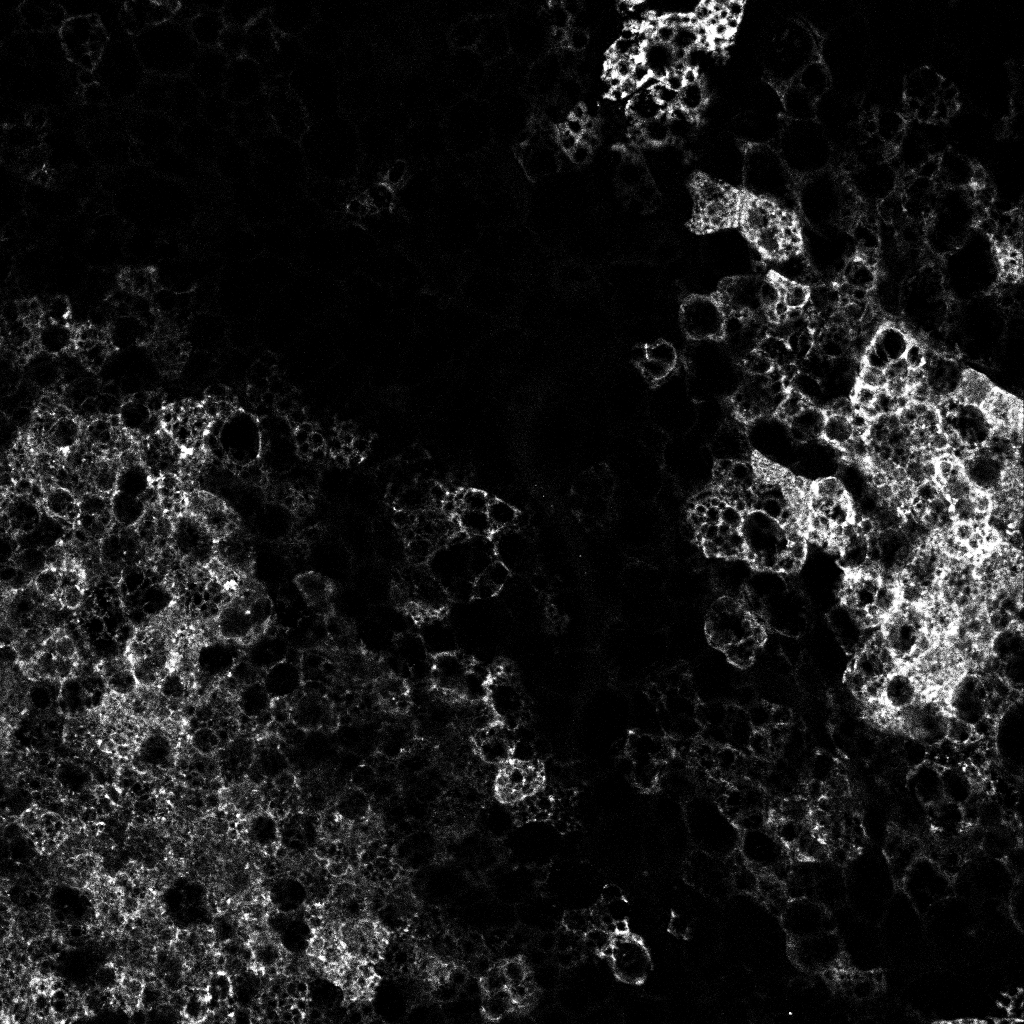

Supplement: Supplementary file 8 — Source Data Fig. 7 [file 44318_2023_8_MOESM8_ESM.zip › Figure 7/7G/confocul iWAT UCP1 LD Nuc PDSS2BKO.tif]

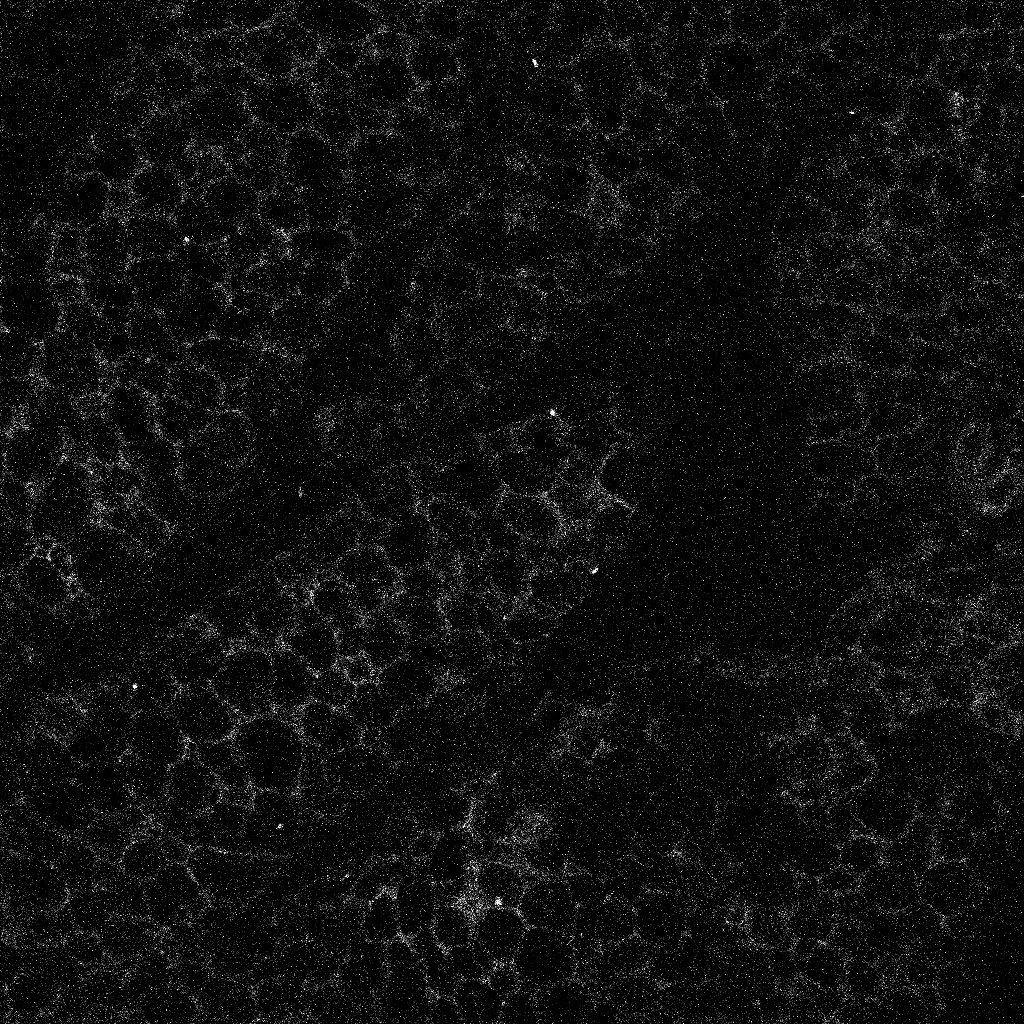

Supplement: Supplementary file 8 — Source Data Fig. 7 [file 44318_2023_8_MOESM8_ESM.zip › Figure 7/7G/confocul iWAT UCP1 LD Nuc PDSS2FL.tif]
