# Supplementary material for: Brown adipose tissue CoQ deficiency activates the integrated stress response and FGF21-dependent mitohormesis
Source: EMBO J. 2024 Jan 11;43(2):2. doi: 10.1038/s44318-023-00008-x (PMC10897314; doi:10.1038/s44318-023-00008-x)
Supplement: Supplementary file 3 — Source Data Fig. 2 [file 44318_2023_8_MOESM3_ESM.zip › Figure 2/2A/README.rtf]

Images taken by confocal microscopy using a Zeiss LSM 880 of murine brown adipocyte cells treated with a vehicle control (CTL) or 4CBA. Mitochondria is pictured in red, lipid droplets are pictured in green and nuclei are pictured in blue.
