# Supplementary material for: Brown adipose tissue CoQ deficiency activates the integrated stress response and FGF21-dependent mitohormesis
Source: EMBO J. 2024 Jan 11;43(2):2. doi: 10.1038/s44318-023-00008-x (PMC10897314; doi:10.1038/s44318-023-00008-x)
Supplement: Supplementary file 3 — Source Data Fig. 2 [file 44318_2023_8_MOESM3_ESM.zip › Figure 2/2D/README.rtf]

Images taken using transmission electron microscopy with the FEI Tecnai 12 120kV TEM (FEI) of murine brown adipocyte cells treated with vehicle control (CTL) or 4CBA. 
