# Supplementary material for: Brown adipose tissue CoQ deficiency activates the integrated stress response and FGF21-dependent mitohormesis
Source: EMBO J. 2024 Jan 11;43(2):2. doi: 10.1038/s44318-023-00008-x (PMC10897314; doi:10.1038/s44318-023-00008-x)
Supplement: Supplementary file 8 — Source Data Fig. 7 [file 44318_2023_8_MOESM8_ESM.zip › Figure 7/7D/README.rtf]

Representative image of inguinal white adipose tissue (iWAT) lobes dissected from floxed control (PDSS2FL) and brown adipose tissue specific PDSS2 knockout (PDSS2BKO) mice.
