# Supplementary material for: Brown adipose tissue CoQ deficiency activates the integrated stress response and FGF21-dependent mitohormesis
Source: EMBO J. 2024 Jan 11;43(2):2. doi: 10.1038/s44318-023-00008-x (PMC10897314; doi:10.1038/s44318-023-00008-x)
Supplement: Supplementary file 8 — Source Data Fig. 7 [file 44318_2023_8_MOESM8_ESM.zip › Figure 7/7G/README.rtf]

Confocal microscopy images taken using a Zeiss LSM 710 microscope of inguinal white adipose tissue (iWAT) sections from floxed control (PDSS2FL) and brown adipose tissue specific PDSS2 knockout (PDSS2BKO) mice. UCP1 is pictured in red, lipid droplets are pictured in green and nuclei are pictured in blue.
