# Supplementary material for: Brown adipose tissue CoQ deficiency activates the integrated stress response and FGF21-dependent mitohormesis
Source: EMBO J. 2024 Jan 11;43(2):2. doi: 10.1038/s44318-023-00008-x (PMC10897314; doi:10.1038/s44318-023-00008-x)
Supplement: Supplementary file 11 — Expanded View Figures [file 44318_2023_8_MOESM11_ESM.pdf]

Expanded View Figures

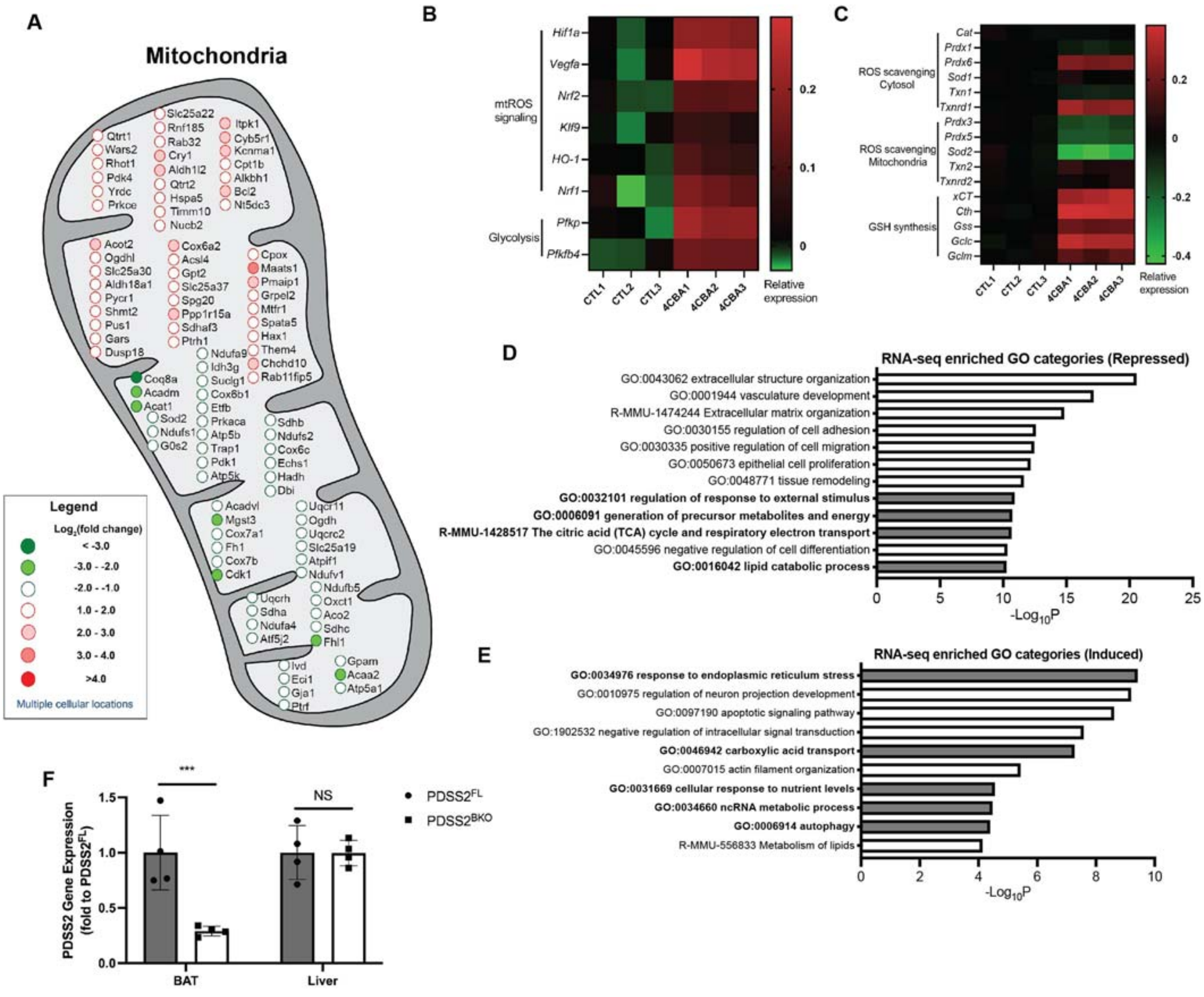

Figure EV1. Enriched genes and pathways in CoQ deficient murine brown adipocytes.

(**A**) Top 50 upregulated and top 50 downregulated mitochondrial genes from RNA sequencing results with *p* value ≤ 0.00418062. (**B**, **C**) Relative expression (log_{10} -transformed RPKM value) of enriched genes in murine brown adipocytes treated with 4CBA (24 h). (**D**, **E**) Gene Ontology (GO) analysis of RNAseq data from 24-h 4CBA treated brown adipocytes. Representative GO categories repressed or induced. (**F**) Validation of tissue specific knockout of PDSS2 via qPCR analysis in BAT and liver of PDSS2 floxed ($PDSS2^{FL}$) or BAT-specific PDSS2 knockout ($PDSS2^{BKO}$) animals, $n = 4$. Results were compared using an unpaired two-tailed Student's *t* test. Significance presented at $^{***}P < 0.001$ compared to controls. Data describes biological replicates. Data information: (**A**, **D**, **E**) The Wald test was used for statistical analysis.

 © The Author(s)

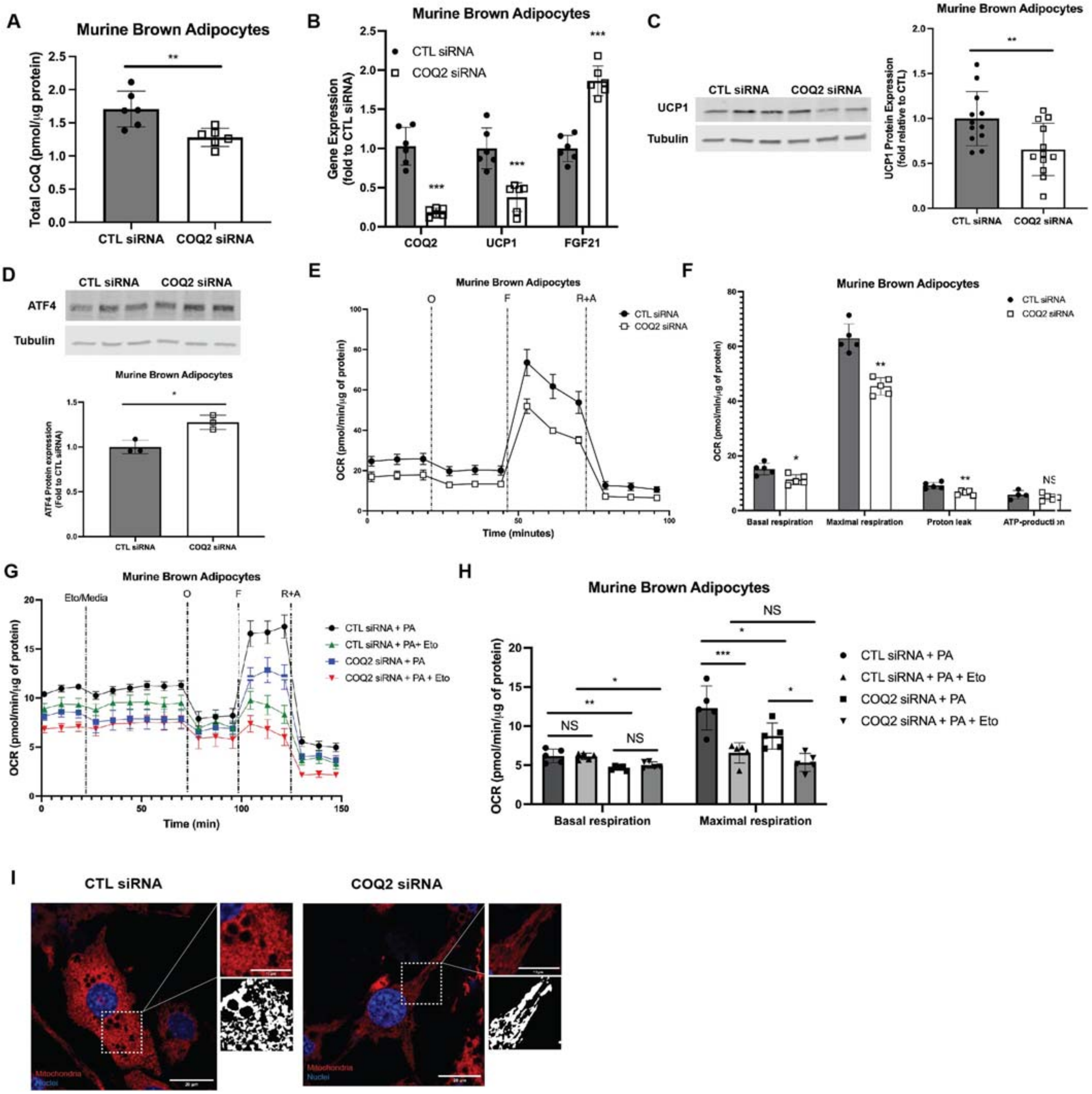

Figure EV2. Genetically induced CoQ deficiency in murine brown adipocytes via knockdown of COQ2.

(**A**) Total CoQ levels, including CoQ_9 and CoQ_{10} isoforms, of murine brown adipocytes transfected with either a scramble siRNA (CTL siRNA) or COQ2 siRNA, $n = 6$. (**B**) Gene expression of CTL siRNA and COQ2 siRNA-treated cells, $n = 5$. (**C**) UCP1 protein expression of CTL siRNA and COQ2 siRNA-treated cells, $n = 12$. (**D**) ATF4 protein expression of CTL siRNA and COQ2 siRNA treated cells, $n = 3$. (**E**) Oxygen consumption rate (OCR) of CTL siRNA and COQ2 siRNA treated cells during mitochondrial stress test, $n = 5$. (**F**) Basal, maximal, protein leak and ATP production respiration of CTL siRNA and COQ2 siRNA treated cells calculated from oxygen trace shown in (**E**), $n = 5$. (**G**) Fatty acid oxidation assay was performed on cells treated with CTL siRNA + palmitate (PA), CTL siRNA + PA + Etomoxir (Eto), COQ2 siRNA + PA and COQ2 siRNA + PA + Eto and OCR was measured, $n = 5$. (**H**) Basal and maximal respiration of the same cells whose oxygen trace is shown in (**E**), $n = 5$. Results were compared using a one-way ANOVA test. (**I**) Brown adipocytes with CTL siRNA or COQ2 siRNA treatment were stained with MitoTracker deep red, and dapi and visualized using the Zeiss LSM880, scale bar = 20 μm. Data information: Data are mean ± SEM. (**A–D**, **F**) Results were compared using an unpaired two-tailed Student's *t* test. Significance presented at *$P < 0.05$, **$P < 0.01$, and ***$P < 0.001$ compared to controls. Data describes biological replicates.

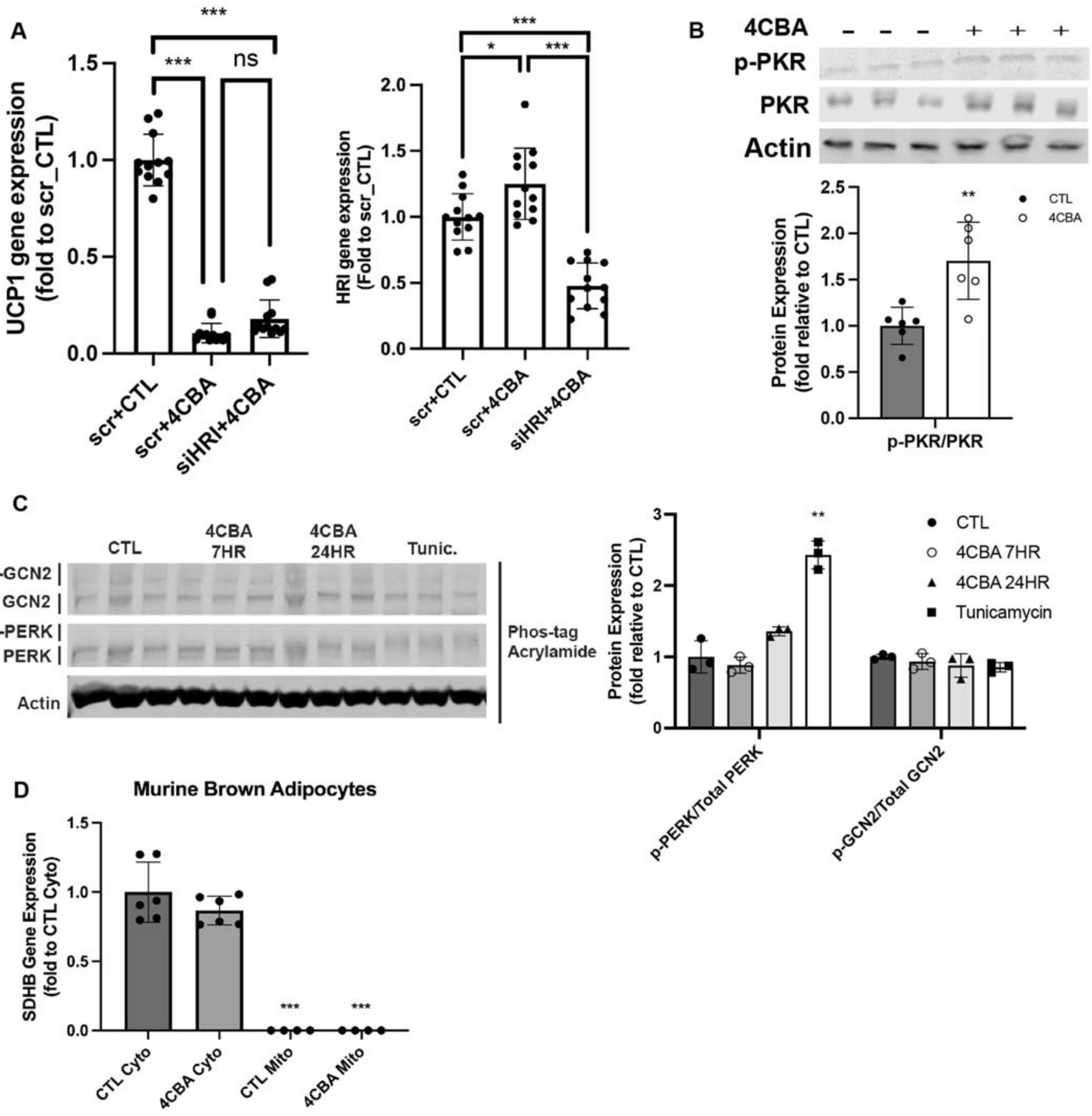

Figure EV3. Targeting stress kinases to study ISR induction.

(**A**) *UCP1* and *HRI* gene expression in brown adipocytes transfected with scramble or HRI siRNA under vehicle control or 4CBA treatment for 24 h, $n = 12$. Pooled data for three repeated experiments. Results were compared using a one-way ANOVA test. (**B**) Western blot analysis of PKR and phosphorylated PKR (Thr451) levels, $n = 6$. (**C**) Phos-tag gel analysis of p-GCN2 and p-PERK compared to total GCN2 and total PERK, $n = 3$. (**D**) SDHB gene expression of cytosolic or mitochondrial cell fractions from vehicle or 4CBA-treated cells, $n = 4$–6. Data information: Data are mean ± SEM. (**B**–**D**) Results were compared using an unpaired two-tailed Student's *t* test. Significance presented at $^{*}P < 0.05$, $^{**}P < 0.01$, and $^{***}P < 0.001$ compared to controls. Data describes biological replicates.

 © The Author(s)

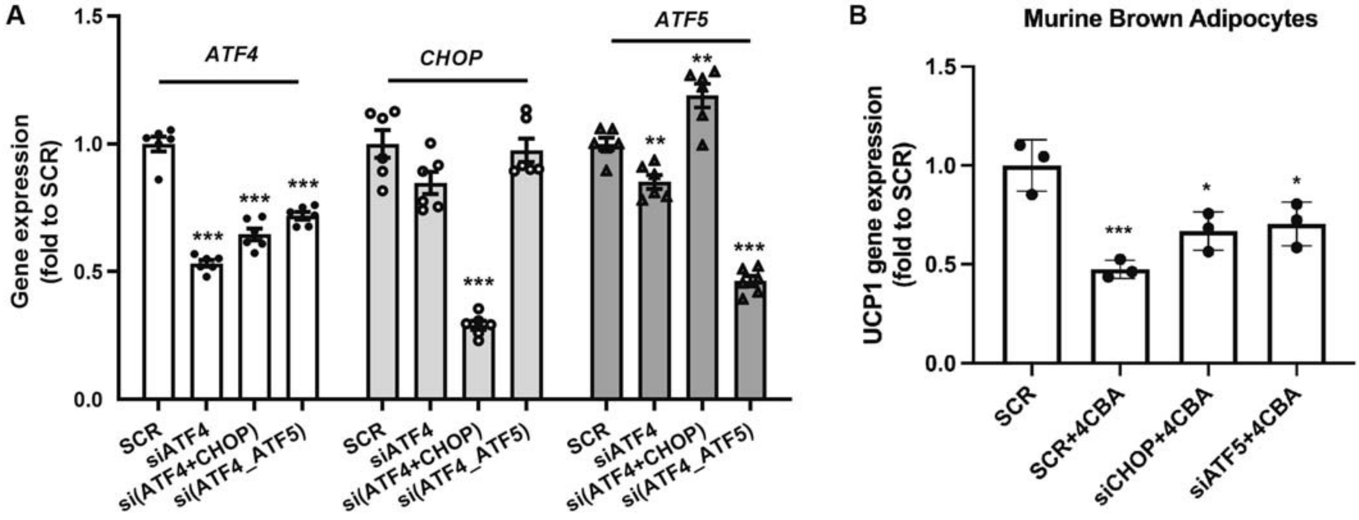

Figure EV4. Targeting ISR-related components.

(**A**) *ATF4*, *CHOP*, and *ATF5* expression in brown adipocytes with 4CBA treatment after indicated siRNA knockdown of target genes (*x*-axis), $n = 6$. Results were compared using an unpaired two-tailed Student's *t* test. (**B**) *UCP1* expression in 4CBA-treated brown adipocyte with siRNA-mediated knockdown of *CHOP* or *ATF5*, $n = 3$. Results were compared using a one-way ANOVA test. Data information: Data are mean ± SEM. Significance presented at $*P < 0.05$, $**P < 0.01$, and $***P < 0.001$ compared to controls. Data describes biological replicates.

© The Author(s)

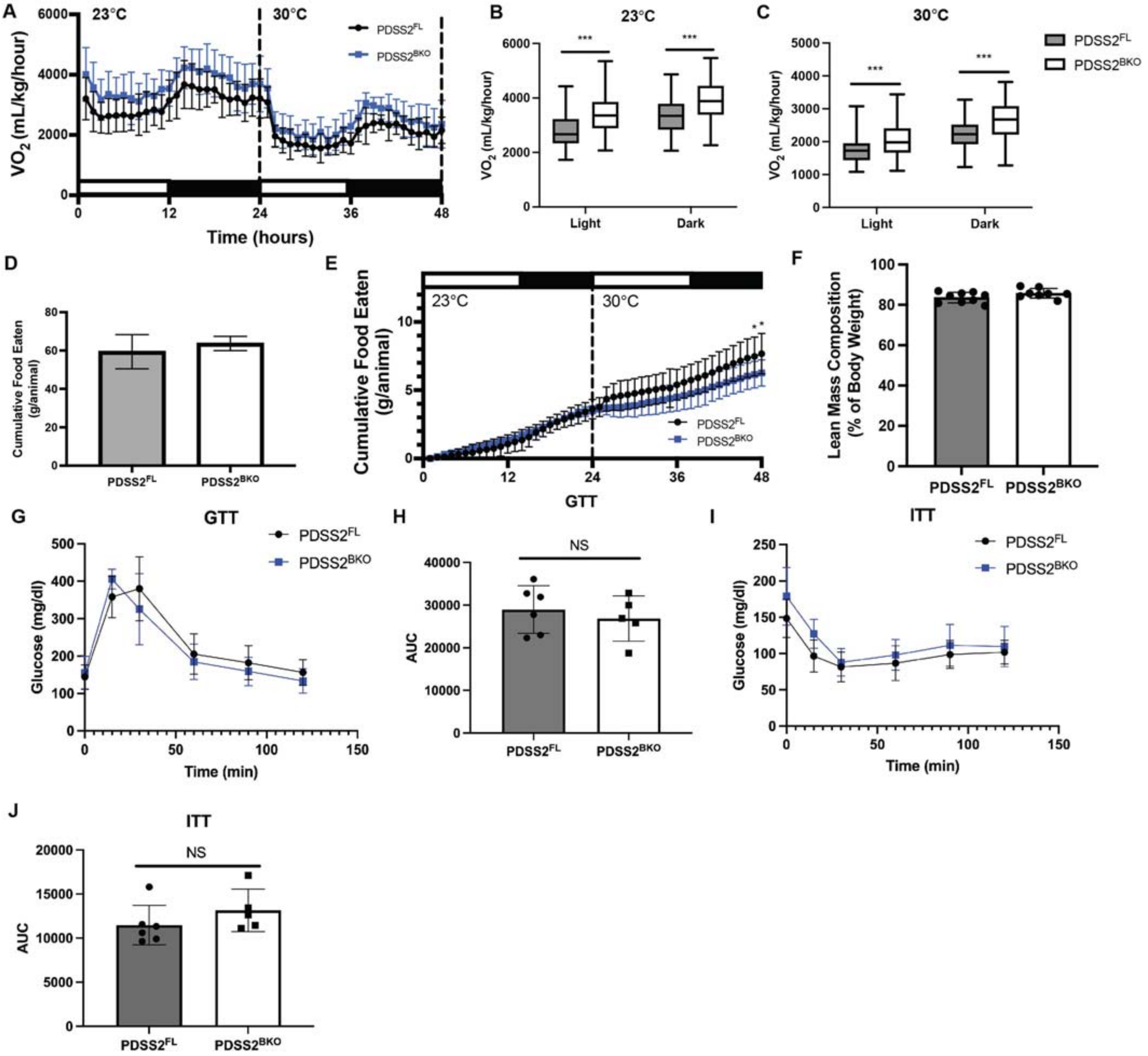

Figure EV5. Metabolic parameters of PDSS2 knockouts.

(**A**) Oxygen consumption rate of PDSS2^{FL} or PDSS2^{BKO} animals was monitored under 23 and 30 °C in real-time in metabolic cages using CLAMS. Data normalized to body weight. (**B**, **C**) Averaged oxygen consumption rate. Centerline represents median and box extends from 25th to 75th percentiles. Data normalized to body weight. (**D**) Food consumption accumulated in three weeks. (**E**) Real-time feeding data from metabolic cage run at 23 and 30 °C. (**F**) Lean mass composition of PDSS2^{FL} and PDSS2^{BKO} animals. (**G**) Glucose tolerance test (GTT) of PDSS2^{FL} and PDSS2^{BKO} animals. 2.0 g/kg body weight glucose dose administered via intraperitoneal (IP) injection. (**H**) Area under curve (AUC) analysis of GTT presented in (**G**). (**I**) Insulin tolerance test (ITT) of PDSS2^{FL} and PDSS2^{BKO} animals. 0.4 IU/kg body weight insulin dose administered via IP injection. (**J**) Area under curve (AUC) analysis of ITT presented in (**I**). Data information. (**A–C**) $n = 10$–11/group. (**D–F**) $n = 8$–9/group. (**G–J**) $n = 5$–6/group. Data are mean ± SEM. Results were compared using an unpaired two-tailed Student's *t* test. Significance presented at $*P < 0.05$, and $***P < 0.001$ compared to controls. Data describes biological replicates.

 © The Author(s)
